# Immune status alters the probability of apparent illness due to dengue virus infection: Evidence from a pooled analysis across multiple cohort and cluster studies

**DOI:** 10.1371/journal.pntd.0005926

**Published:** 2017-09-27

**Authors:** Hannah E. Clapham, Derek A. T. Cummings, Michael A. Johansson

**Affiliations:** 1 Department of Epidemiology, School of Public Health, Johns Hopkins University, Baltimore, MD, United States of America; 2 Dengue Branch, Division of Vector-Borne Diseases, Centers for Disease Control and Prevention, San Juan, PR; 3 Center for Communicable Disease Dynamics, Harvard TH Chan School of Public Health, Boston, MA, United States of America; University of North Carolina at Chapel Hill, UNITED STATES

## Abstract

Dengue is an important vector-borne pathogen found across much of the world. Many factors complicate our understanding of the relationship between infection with one of the four dengue virus serotypes, and the observed incidence of disease. One of the factors is a large proportion of infections appear to result in no or few symptoms, while others result in severe infections. Estimates of the proportion of infections that result in no symptoms (inapparent) vary widely from 8% to 100%, depending on study and setting. To investigate the sources of variation of these estimates, we used a flexible framework to combine data from multiple cohort studies and cluster studies (follow-up around index cases). Building on previous observations that the immune status of individuals affects their probability of apparent disease, we estimated the probability of apparent disease among individuals with different exposure histories. In cohort studies mostly assessing infection in children, we estimated the proportion of infections that are apparent as 0.18 (95% Credible Interval, CI: 0.16, 0.20) for primary infections, 0.13 (95% CI: 0.05, 0.17) for individuals infected in the year following a first infection (cross-immune period), and 0.41 (95% CI: 0.36, 0.45) for those experiencing secondary infections after this first year. Estimates of the proportion of infections that are apparent from cluster studies were slightly higher than those from cohort studies for both primary and secondary infections, 0.22 (95% CI: 0.15, 0.29) and 0.57 (95% CI: 0.49, 0.68) respectively. We attempted to estimate the apparent proportion by serotype, but current published data were too limited to distinguish the presence or absence of serotype-specific differences. These estimates are critical for understanding dengue epidemiology. Most dengue data come from passive surveillance systems which not only miss most infections because they are asymptomatic and often underreported, but will also vary in sensitivity over time due to the interaction between previous incidence and the symptomatic proportion, as shown here. Nonetheless the underlying incidence of infection is critical to understanding susceptibility of the population and estimating the true burden of disease, key factors for effectively targeting interventions. The estimates shown here help clarify the link between past infection, observed disease, and current transmission intensity.

## Author summary

Dengue disease severity is known to vary widely from the very severe to asymptomatic. There is a wide range of estimates of how many infections result in each of these outcomes. It is known that after a first infection the outcome of a second infection with a different serotype varies over time, but this has not been taken into account in these previous estimates. In this paper, we use modelling methods, combined with information from published dengue research in which individuals are followed over time, to estimate the proportion of infections that result in symptoms at different times after infection. We estimated the proportion of infections that are symptomatic for first infections as 0.18 (95% Credible Interval, CI: 0.16, 0.20), 0.13 (95% CI: 0.05, 0.17) for individuals infected in the year following a first infection and 0.41 (95% CI: 0.36, 0.45) for those experiencing secondary infections after this first year. The estimates here will help understand how cases relate to underlying transmission, which is vital for understanding how much of the population are susceptible to infection and for effectively targeting interventions.

## Introduction

Dengue is an important vector-borne disease found across much of the world [1]. The four dengue virus serotypes have complex immunological interactions whereby infection with one serotype is thought to lead to a period of short-term protective immunity against all serotypes, followed by a period in which infection with a different serotype is more likely to result in severe disease [2]. At each of these immune stages a large proportion of infections result in few or no symptoms [3], while others result in severe illness. It is well documented that a second infection with a heterologous serotype after the period of protection is more likely to result in severe disease [4], but less clear whether this second infection is also more likely to be apparent than a primary infection. The first evidence of cross-protection came from Sabin’s studies of experimental dengue infections showing protection against virus and/or symptoms upon experimental infections after a previous dengue infection [5,6]. Sabin’s studies showed some protection against symptomatic infection up to nine months after infection (the longest time after first infection that was tested).

Previous estimates of the proportion of dengue infections that are apparent have come from cohort studies and cluster studies. Cohort studies follow the same individuals over time, usually recording antibody titres at consistent intervals (months to years), as well as recording whether individuals experienced a symptomatic dengue infection in these intervals. Cluster studies focus on testing individuals living in close proximity to known dengue cases and recording whether those individuals have experienced disease. An asymptomatic or inapparent infection is usually defined as a substantial rise in antibody titres between two measurements in a participant not experiencing symptoms. Symptomatic or apparent infections are infections concurrent with compatible symptoms, with the infection usually virologically confirmed. A recent review found that inapparent proportion estimates varied from 8–100% across relevant studies [3]. The review also highlighted a positive correlation between the proportion of cases which are inapparent, and the incidence in the previous year. This relationship, which has also been shown in some of the individual papers [7], was thought to be linked to the period of short-term cross-protective immunity. Various investigators also suggest that the proportion of inapparent infections varies between locations due to virus serotype, genotype or age of host [3,8]. Because these different factors are sources of variation, estimating the overall proportion of infections resulting in symptomatic disease, or considering the impact of each of these factors in turn is challenging. We therefore conducted an analysis to estimate the apparent proportion in each of these studies separately and together, in a pooled analysis. We estimated the proportion of infections that were apparent for individuals in different immunological phases and upon infections with the different serotypes.

## Materials and methods

We performed a search in PubMed with the terms dengue cohort and dengue cluster study. We also searched the references of a recent review of dengue inapparent infections [3]. Papers were included in the analysis if it was possible to determine a number of inapparent infections and a number of apparent infections occurring in a single population and time frame. For cohort studies, we also required that apparent infections were laboratory confirmed at the time of illness to ensure that symptoms were associated with the DENV infection and not another acute febrile illness (confirmation methods are included in Table 1). For cluster studies, we required that sufficient information be provided that the index cases could be removed from the analysis. Including the index case, which is by definition apparent, introduces a bias in the analysis.

**Table 1:** Studies from which data was extracted for the analysis. HI: Haemagglutination inhibition, PRNT: Plaque reduction neutralisation titre, ELISA: Enzyme linked immunosorbent assay.

Study	Study type	Age group (yrs)	Inapparent infection identification	Apparent case identification	Serotype data in paper	Analysis
Philippines[13]	Cohort	0.5–85	HI: 4-fold increase	Fever AND RT-PCR: IgM positive or 4-fold IgG increase	Symptomatic infections only	A
Brazil, Colombia, Puerto Rico (A) and Mexico [14]	Cohort	9–16	ELISA: IgG seroconversion (primary only)	2 days fever AND RT-PCR: positive OR ELISA: IgM positive or 4-fold IgG increase	None	A
Nicaragua [15]	Cohort	2–9	HI: 4-fold increase	Fever AND RT-PCR: positive OR ELISA: IgM positive or 4-fold inhibition increase	Apparent infections only	A, B, D
Sri Lanka [16]	Cohort	0–12	ELISA: IgG seroconversion (primary only), PRNT: 2-fold increase (secondary)	Fever AND RT-PCR: positive OR ELISA: IgM positive or 4-fold IgG increase	Apparent infections only	A, D
Peru [17]	Cohort	0–75	PRNT: seroconversion	Fever and one other dengue symptom AND RT-PCR: positive OR ELISA: 4-fold IgM increase	All infections	A, D
Vietnam [18]	Cohort	2–15	ELISA: IgG seroconversion (primary only)	Fever and suspected dengue or viral disease AND RT-PCR: positive OR ELISA: IgM positive or 4-fold IgG increase	Apparent infections only	A, B, D
Thailand (A) (Bangkok) [19]	Cohort	4–16	HI or PRNT: seroconversion (primary) or 4-fold increase (secondary)	2 day school absence for fever AND ELISA: 4-fold IgM increase OR HI or PRNT: seroconversion (primary) or 4-fold increase (secondary)	Apparent infections only	A
Thailand (B) (Kamphaeng Phet) [7]	Cohort	4–15	HI: 4-fold increase AND PRNT: 4-fold increase	2 day school absence OR fever AND ELISA: IgM positive or 4-fold IgG increase	Apparent infections only	A, B, D
Puerto Rico (B) [20]	Cohort	10–18	PRNT: 4-fold increase	Fever AND RT-PCR: positive OR ELISA: IgM positive	Apparent infections only	A
Thailand (B) [7]	Cluster	1–15	ELISA: IgM positive OR 4-fold IgG increase	Any symptoms AND ELISA: IgM positive or 4-fold IgG increase	Index case only	C
Vietnam [21]	Cluster	5–55	ELISA: seroconversion OR RT-PCR: positive OR NS1: positive	Fever AND ELISA: seroconversion OR RT-PCR: positive OR NS1: positive	Index case only	C
Nicaragua [22]	Cluster	2–60+	ELISA: seroconversion HI: 4-fold increase	WHO definition of DF or undifferentiated fever AND ELISA: IgM seroconversion OR HI: 4-fold increase	All infections	C
Indonesia [23]	Cluster	9–55	RT-PCR: positive HI: 4-fold increase	Fever AND RT-PCR: positive OR ELISA: seroconversion	All infections	C

### Model

We made several key assumptions about the infection risk and the risk of symptomatic disease in order to formulate our model. First, we assumed that for a given study (j) and time period (i), the infection risk (ρ_i, j_) was equal for immunologically naïve individuals and for individuals with a previous infection. We then assumed that the proportion of infections resulting in symptomatic disease (γ) for each infection group (primary: γ’, or secondary: γ”) was equivalent across all time periods and studies (though we also made study-specific estimates). We then used study data to identify for each study and year: (1) the number of subjects with no previous dengue exposure (N_naïve, i, j_) who were susceptible to primary infection (indicated as IgG negative), (2) the number of subjects with previous dengue exposure (N_prev, i, j_) (IgG positive), (3) the number of inapparent infections (seroconversion), O’_inapp, i, j_ and O”_inapp, i, j_, for primary and secondary infections, respectively, and (4) the number of symptomatic infections (acute seroconversion or detection of viral RNA), O’_app, i, j_ and O”_app, i, j_ for primary and secondary infections, respectively. For each class of observation (O), we assumed that number of observed infections came from a binomial distribution with the respective population of each group from that the study, N, and a year and location-specific probability of infection (ρ_i, j_) and the group-specific probability of having apparent disease (γ’ or γ”):

$$\begin{array}{l} O({\mathrm{app}}_{i}^{'})∼Binomial(N_{naive},\rho_{i}\gamma_{}^{'})\\ O({\mathrm{app}}_{i}^{'})∼Binomial(N_{naive},\rho_{i}(1-\gamma_{}^{'}))\\ O({\mathrm{inapp}}_{i}^{''})∼Binomial(N_{prev},\rho_{i}\gamma_{}^{''})\\ O({\mathrm{inapp}}_{i}^{''})∼Binomial(N_{prev},\rho_{i}(1-\gamma_{}^{''})) \end{array}$$

We extended the model to include a period of possible altered immunity (cross-immunity) in the year following infection. This was modelled by including a third susceptibility group N_rec_,_i,j_, which was individuals who had experienced an infection in the preceding cohort year, and allowing the model to fit a different probability of apparent infection during this period (γ_rec_,_i,j_).

Each model was fit to the data in a Bayesian framework using rStan [9]. We assumed that the γ and ρ parameters were unknown and assigned each a naive beta prior (α = 1 and β = 1). The model code is available in the supplementary materials (S1 File).

## Results

The PubMed search for “dengue cohort” returned 357 papers. The references for Grange et al. added an additional 3 papers and 2 more were discovered through personal communication. Of these, 9 papers on 12 different cohorts contained sufficient published information on both apparent and inapparent infections in the same cohort. The search for “dengue cluster study” returned 180 papers, with 4 papers on 4 different cluster studies containing enough information on the number of non-index cases in the cluster during the follow up period. Table 1 shows the studies included, study type, how infections and cases were determined in each study, and whether the publication included serotype information. Several studies included in previous analyses of the apparent proportion [3] were excluded here because of a lack of laboratory confirmation at the time of illness [10–12].

We present the results of four analyses in this manuscript. For Analysis A, we estimated the apparent proportion without controlling for serotype or temporary cross-protection for each of 12 studies individually and for all 12 studies together. For Analysis B, we used multi-year cohort studies (3) to estimate apparent proportions for secondary infection in two different groups: (1) individuals with recent primary infection (the previous year), who may experience cross-protection; and (2) individuals with more distant primary exposure (more than one year). For Analysis C, we estimated the apparent proportion using only cluster studies (4 studies). Finally using the cohort studies, for Analysis D, we estimated serotype specific apparent proportions for primary and secondary infections (5 studies). The final column of Table 1 indicates which studies were included in each analysis.

### Primary and secondary infections across all cohort studies (Analysis A)

**Fig 1:**
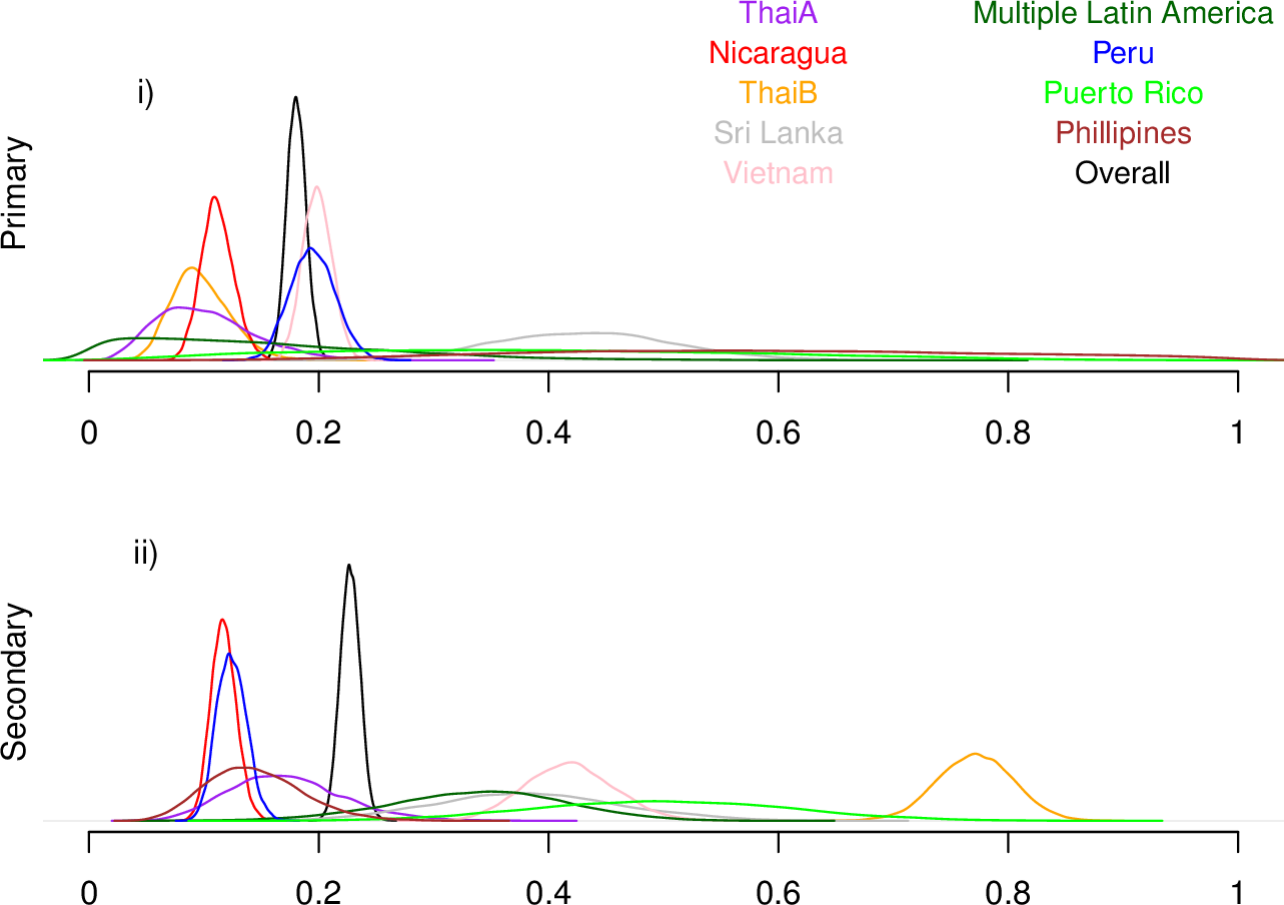
Estimated probability of apparent disease given infection by study. Probability densities of estimates for the apparent proportion in primary (i) and secondary (ii) infection for each study (Analysis A for each study separately).

For each individual cohort study, the estimated apparent proportion for secondary infection was close to or slightly higher than the estimate for primary infection (Fig 1). There were two exceptions; in Peru the secondary estimate was lower than the primary and in Thailand-KPP, the secondary estimate was substantially higher than the primary.

**Fig 2:**
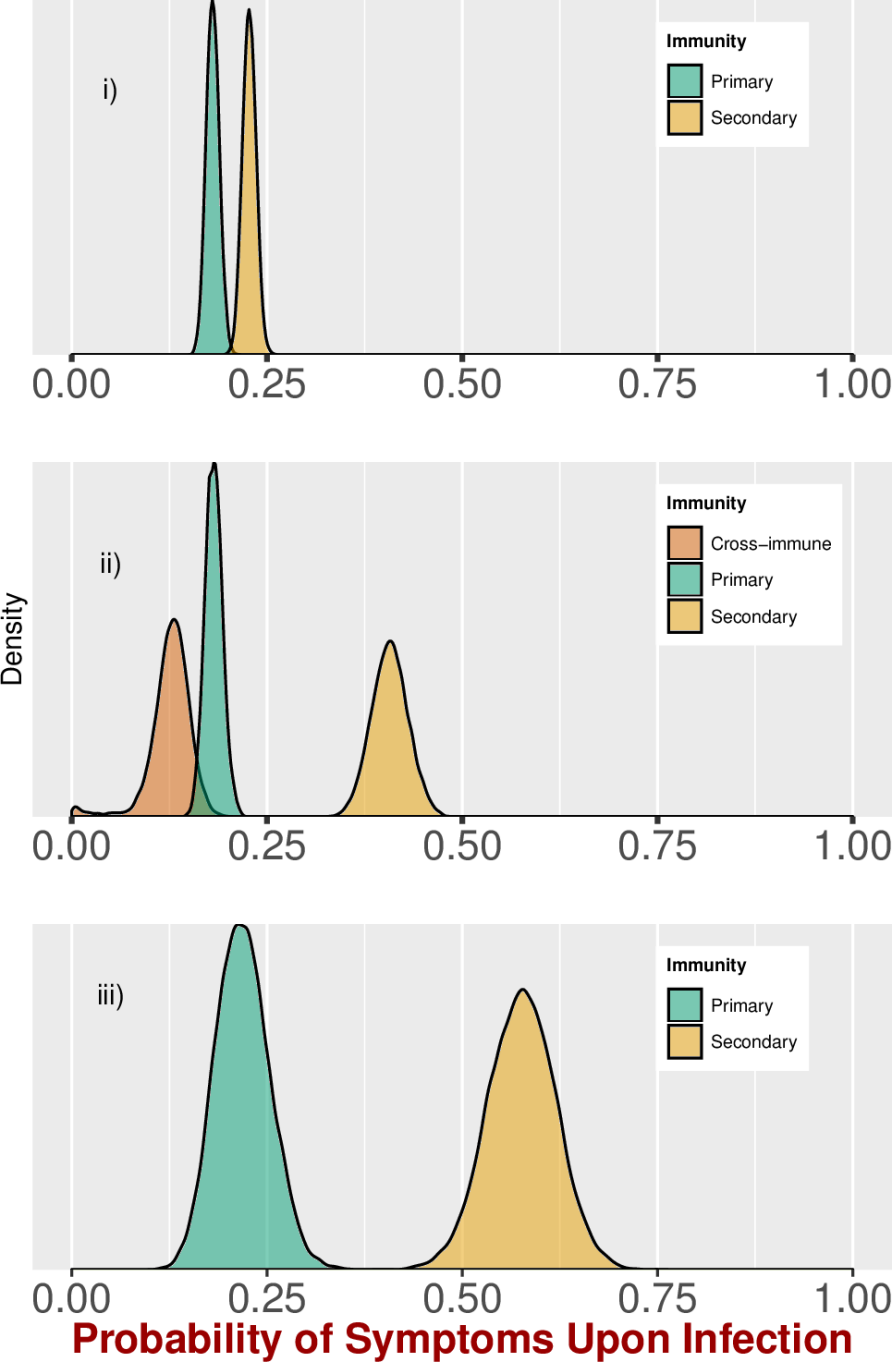
Overall estimated probability of apparent disease given infection. (i) Probability densities of estimates of the apparent proportion in primary and secondary infections from cohort studies (Analysis A). (ii) Probability densities of estimates including a period of cross-immunity (Analysis D). (iii) Probability densities of estimates from cluster studies (Analysis C). For (i) and (iii) estimates for primary infection shown in green, secondary infection in orange and for (ii) estimates for primary infection shown in green, secondary infections in the year after infection in brown and secondary infections in the subsequent years in orange.

In the analysis including all 12 cohort studies with shared parameters for the proportion of infections experiencing disease and different local infection risks, we estimated an overall apparent proportion that was significantly higher for secondary infections (0.24, 95% Credible Interval, CI: 0.22, 0.26) than for primary infections (0.18, 95% CI: 0.16, 0.19) (Fig 2i).

**Fig 3:**
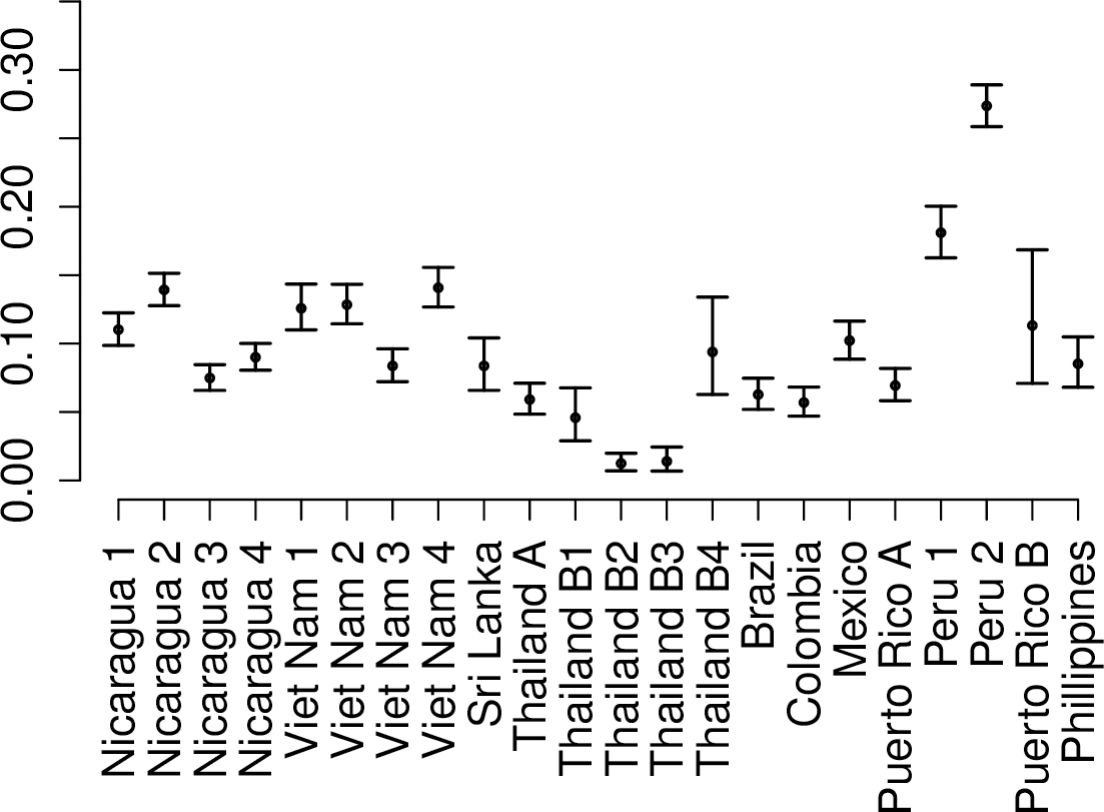
Estimated risk of infection from cohort studies. The yearly probability of infection in the cohort studies for each study year (from Analysis A all studies together).

The modelling framework incorporated local data on primary and secondary cases with apparent and inapparent infection as well as global parameters for the probability of apparent disease in primary and secondary infection to estimate transmission intensity (Fig 3). Those estimates showed high variability in incidence of infection between locations and between years in the same cohorts.

### Estimates including a period of cross-protection (Analysis B)

Three studies had data collected across multiple years and therefore sufficient information to estimate the impact of short-term cross-protective immunity. The estimated apparent proportion for primary infections among these studies was similar to Analysis A, 0.18 (95% CI: 0.16, 0.20) (Fig 2ii). Among secondary infections, however, we estimated that infections within one year of the primary infection had an apparent proportion less than or equal to that of primary infections, 0.13 (95% CI: 0.05, 0.17), indicating short-term protection from apparent disease. On the other hand, secondary infections after the cross-immune period had a substantially higher symptomatic proportion, 0.41 (95% CI: 0.36, 0.45), (Fig 2ii).

### Estimates from cluster studies (Analysis C)

**Fig 4:**
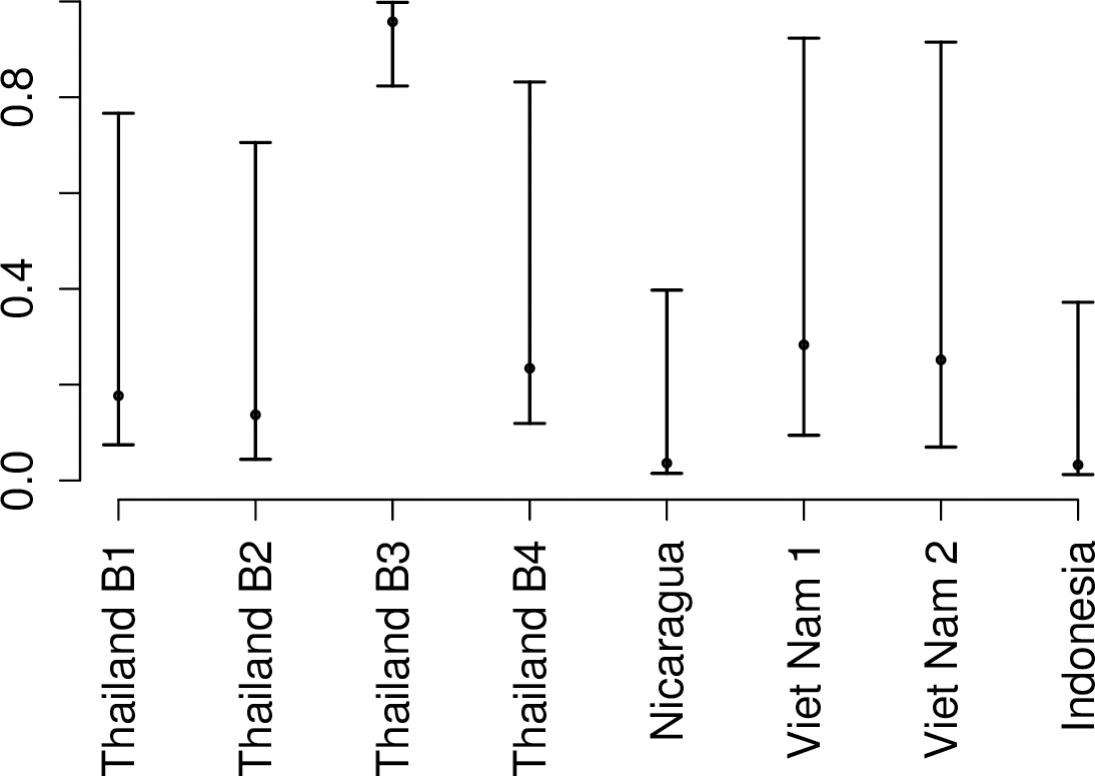
Estimated risk of infection from cluster studies. The probability of infection in the time of follow up for those in the cluster around an index case (Analysis C).

We then estimated the apparent proportion using data exclusively from the four cluster studies that included sufficient data. The primary infection estimate of 0.22 (95% CI: 0.15, 0.29) from these data was similar to that from the cohort studies (Analyses A and B). However, the secondary infection estimate of 0.57 (95% CI: 0.49, 0.68) was significantly higher than the general estimate for all cohorts (Analysis A) and closer to the estimates for secondary infections more than one year after primary infection (Analysis B) (Fig 2). As for the cohort studies, we simultaneously estimated the probability of individuals in the cluster being infected in the follow up period (Fig 4). The mean estimates were generally higher that estimates for cohort studies, yet they generally had large credible intervals due to small sample sizes.

### Serotype-specific estimates (Analysis D)

**Fig 5:**
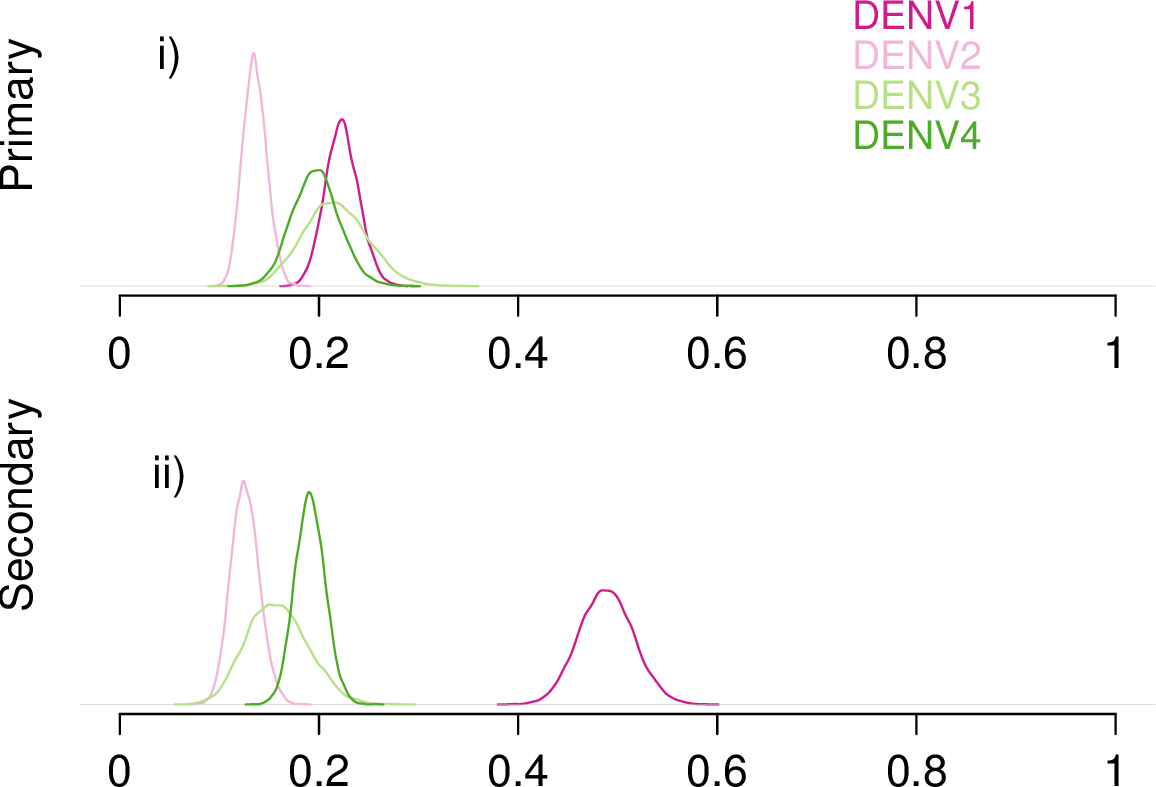
Estimated probability of apparent disease given infection by serotype. Probability densities of estimates for the apparent proportion in primary (i) and secondary (ii) infection across serotypes (Analysis B).

Finally, we estimated serotype-specific apparent proportions for the five cohort studies that had sufficient data available (Fig 5i and 5ii). For primary infections, each serotype overlapped substantially. For secondary infections, there appeared to be some differences, most notably with DENV1. However the data for DENV1 came from a single study, the Thailand-KPP study, and thus only indicate that a high proportion of infections resulted in apparent disease in that cohort with that genotype at that time and place. The result is therefore not generalizable for other locations and times where circulating genotypes and infection histories may be very different. Likewise, the DENV3 and DENV4 estimates were based on data from a single study in Peru. Only DENV2 data came from multiple locations. So although we were able to fit this model, the data were too limited to generate generalizable results.

## Discussion

We developed a statistical framework to assess the proportion of dengue virus infections that result in apparent disease and how that proportion depends on immune status. This framework allowed us to assess this proportion across different geographical areas, study types, and transmission intensities. The most comprehensive data, from multi-year cohort studies predominantly of children, showed that approximately 18% of primary infections experienced apparent disease. This proportion remained low, approximately 13%, for infections in the year following, but then increased substantially for secondary infections beyond the first year to approximately 45%. The other data analysed substantiated these differences: in individual cohort studies, secondary infections tended to have higher apparent proportions; across 12 cohort studies, the average estimated apparent proportion was significantly higher for secondary infections; and in cluster studies, the difference was even more pronounced.

This finding substantiates evidence from individual cohort studies that have shown that the apparent proportion varies by year and that this variation is related to the incidence of infection in the previous year [3,24,25]. In contrast to previous work, we have for the first time used data from multiple cohorts to explicitly quantify those probabilities and how they change over time related to the local history of dengue virus transmission. This finding has important implications for estimating the force of infection in locations where only data on apparent infections are available, as the relationship between that data and the incidence of infection changes based on immunological history. For example, after a major outbreak, clinical masking of transmission may occur as secondary infections are less likely to be apparent when many people have temporary cross-protective immunity. On the other hand, extensive transmission several years later may appear as a much larger epidemic due to the increased risk of apparent disease in secondary infections several years after primary infection. The finding of the difference in the probability of apparent disease during the cross-protective immune period compared to other periods is striking. This finding is, however, in agreement with other findings showing a relationship between increased cases in one year with an increased inapparent proportion in the next year [24]. While our findings suggest that primary infection confers some short-term protection against apparent disease upon secondary infection, it is unclear how this protection alters the immune response to a secondary infection within that time period and how that may affect immunity to future infections.

Grange et al. [3] previously reviewed most of the studies analysed here. Without considering specific infection histories and the strength of data across locations, estimates of the proportion of infections that were apparent across these studies ranged from 0% to 92% for both primary and secondary infections (presented in the paper as the inapparent proportion). By considering the role of immunity, the incidence of infection, and a shared probability of apparent disease, we were able to further resolve this data and show a significant different between primary and secondary infections. Our estimates are also similar to the infection to symptomatic ratio of 4.3 used by Bhatt et al. in generating global estimates of disease burden. Their ratio is roughly equal to 18% of infections being apparent, very close to what we estimated for primary infections, however lower than our estimates for secondary infections after the cross immune period. This difference could importantly change the estimates of the number of clinically apparent dengue cases.

Using data from cluster studies, we estimated higher apparent proportions for both primary and secondary infections. It is possible that cluster studies are better at capturing milder infections than cohort studies as there may be increased effort to identify illness and follow up tends to happen over a shorter time period when recall may be better. Indeed, Grange et al. [3] noted that cases in cluster studies compared to the cohorts were milder. It is also possible that previous exposure plays a role here. Given similar infection risk, apparent cases (i.e. index cases for clusters) are less likely to appear in areas with recent transmission and more cross-immunity. Therefore, cluster studies may be biased towards areas with higher risk of apparent disease specifically due to transmission history. This is supported by our finding that the cluster-based apparent proportion for secondary infections was close to our cohort-based estimate only after accounting for cross-protective immunity. The clusters are also different from the cohorts because they routinely include adult cases, which may have a different probability of being apparent compared to children, even given similar infection history. Although further work is needed to help consider these biases, the general results are largely in agreement, with an apparent proportion on the order of 20% for primary infections and 2–3 times higher for secondary infections.

The analytical approach used here enabled simultaneous estimation of multiple unknowns (infection risk and multiple apparent proportions) in a context of limited outcome information. We drew on data from different locations where infection history and risk may drive differences in apparent incidence. As suggested here, the apparent proportion may actually be quite similar across locations, despite possibly appearing different due to different histories of recent exposure. Combining data from multiple locations has great benefit in increasing overall sample size and generalizability and allowed us to disentangle the effects of each covariate. An additional benefit of this method is that we were able to estimate the probability of infection over the study period, as has been estimated before [26], but now incorporating shared information about the probability of apparent disease. Furthermore, this approach allowed us to directly compare the probability of infection in cluster studies, which had more uncertainty but indicated that incidence was likely higher. Indeed transmission is likely to be more intense on hyper-local scales with known transmission, as in the clusters.

This analysis represents an important first step towards aggregating knowledge of dengue transmission and disease dynamics globally. This work could be further extended to estimate the contribution of other important factors such as age; available cohort data largely focuses on children. Additional data would also allow assessment of possible differences between serotypes. The current serotype-specific estimates were mainly derived from information from one study per serotype, limiting our ability assess the possibility of serotype-specific effects. Also, variations in the way infections were detected and confirmed (e.g. case definitions, follow-up methods, assay or change in titre) across studies could be better controlled for with the individual level data. These differences may contribute to the variability in the apparent proportion estimates for each study. We have also not explicitly addressed third and fourth infections, which likely also exhibit different patterns. Indeed, some of the infections considered as secondary infections here may have been third or fourth infections, as noted particularly in Peru [17]. Their inclusion may influence the relatively unique results shown for Peru. Though the current assumption is that the probability of any infection is the same in both primary and secondary infections, with the individual level data it may be possible to estimate probabilities of infection separately for primary and secondary cases in cohorts. Differences in the probabilities of infection for primary and secondary cases will give us information on whether exposure risk is different between those groups due to heterogeneous transmission risk and if susceptibility to infection is also changed in the short- or long-term after primary infection. The methods we developed here could also be extended to include additional types of data allowing us to estimate the proportion in different disease severity categories (e.g. severe or hospitalised), which is important to both the health impact and the ability of surveillance systems to detect dengue [27].

Only by drawing on detailed data across multiple years and populations experiencing different infection histories were we able to make general inferences about the relationship between the proportion of infections that are apparent and immune status. These estimates show a clear difference in the apparent proportion between primary and secondary infections, and are helpful for understanding the relationship between the incidence of dengue virus infection and the incidence of disease. For example, even in the presence of an effective intervention, the incidence of disease may increase as there may be an increased delay between primary and secondary infection [28]. Conversely, an ineffective intervention may appear effective if implemented after a major epidemic when cross-immunity may be high. Because the apparent proportion is the key link between infection risk and the observation of disease, a better understanding of this relationship is important for designing, implementing, and evaluating interventions and understanding the dynamics of dengue globally.

## Supporting information

S1 File The supplement contains the code for running the models in the paper in R with rstan. (PDF)
