# Supplementary material for: Immune status alters the probability of apparent illness due to dengue virus infection: Evidence from a pooled analysis across multiple cohort and cluster studies
Source: PLoS Negl Trop Dis. 2017 Sep 27;11(9):e0005926. doi: 10.1371/journal.pntd.0005926 (PMC5633199; doi:10.1371/journal.pntd.0005926)
Supplement: S1 File — (PDF) [file pntd.0005926.s001.pdf]

Code for fitting all the cohorts at the same time

```
#install.packages("rstan", repos = "https://cloud.r-project.org/", dependencies=TRUE)
library(rstan)


asymptomatic_code_cohorts <- '
data {

### Iquitos
int<lower=0> s_iq; // number of serotypes## we have D3 and D4 in this paper
int inappsec_iq[s_iq]; // number of secondary infections that are asymptomatic
int appsec_iq[s_iq]; // number of secondary infections that are symptomatic
int inappprim_iq[s_iq]; // number of primary infections that are asymptomatic
int appprim_iq[s_iq]; // number of primary infections that are symptomatic

## we only have this for DENV4 we have estimated for D3
int Nsec_iq[s_iq];  //each age number ready for secondary infections
int Nprim_iq[s_iq];  //each age number ready for primary infections


### Lat Am
int <lower=0> y_la; // the number of years for which we have data - here 4- actually dif places, but I
think OK
int inapp_la[y_la]; // number of primary infections that are asymptomatic each year
int app_la[y_la]; // number of primary infections that are symptomatic each year
int Nsec_la[y_la];  //number at each age who has already been infected at least once
int Nprim_la[y_la];  //number at each age who have not already been infected at least once (need to
calculate before we put it in..)

### Nicaragua
int <lower=0> y_nic;
int inapp_prim_nic[y_nic];
int app_prim_nic[y_nic];
int inapp_sec_nic[y_nic];
int app_sec_nic[y_nic];
int Nprim_nic[y_nic];
int Nnotprim_nic[y_nic];

## Long Xuyen
int <lower=0> y_lx;
int app_prim_lx[y_lx];
int inapp_prim_lx[y_lx];
int app_sectot_lx;
int Nprim_lx[y_lx];
```

```
int Nsec_lx[y_lx];

## Burke

int inapp_prim_bur;
int inapp_sec_bur;
int app_prim_bur;
int app_sec_bur;
int Nprim_bur;
int Nsec_bur;

## Sri Lanka

int inapp_prim_sr;
int inapp_sec_sr;
int app_prim_sr;
int app_sec_sr;
int Nprim_sr;
int Nsec_sr;

## KPP1

int <lower=0> y_kpp1;
int app_prim_eachyear_kpp1[y_kpp1];
int app_sec_eachyear_kpp1[y_kpp1];
int inapp_eachyear_kpp1[y_kpp1];
int Ntoteachyear_kpp1[y_kpp1];
int Nprim_kpp1;
int Nsec_kpp1;
int Npriminit_kpp1;
int Nsecinit_kpp1;

## Puerto Rico Arguello
int app_prim_pr;
int inapp_prim_pr;
int app_sec_pr;
int inapp_sec_pr;
int Nprim_pr;
int Nsec_pr;

## Phillipines
int app_prim_ph;
int app_sec_ph;
int inapp_ph;
int Nprim_ph;
int Nsec_ph;
```

```
}

parameters {

real <lower=0, upper=1> lambda_iq[s_iq];  // a lambda for each serotype, this is changed to annual for the results to be comparable to the other studies
real <lower=0, upper=1> lambda_la[y_la];
real <lower=0, upper=1> lambda_nic[y_nic];
real <lower=0, upper=1> lambda_lx[y_lx];
real <lower=0, upper=1> lambda_bur;
real <lower=0, upper=1> lambda_sr;
real <lower=0, upper=1> lambda_kpp1[y_kpp1];
real <lower=0, upper=1> lambda_pr;
real <lower=0, upper=1> lambda_ph;
//  no age, no serotype, just primary and secondary
real<lower=0,upper=1> gammaprim;
real<lower=0,upper=1> gammasec;


}
transformed parameters {

### Iquitos
real pappprim_iq [s_iq];// the probabilities of each serotype  being app for prim
real pinappprim_iq [s_iq] ; // the probabilities of each serotype  being inapp for prim
real pappsec_iq [s_iq];// the probabilities of each serotype  being app for sec
real pinappsec_iq [s_iq] ;// the probabilities of each serotype and age being inapp for sec


### Lat Am
real  papp_la[y_la] ;// the probabilities of each year being app
real  pinapp_la[y_la]; // the probabilities of each year being inapp

### Nicaragua
real pinapp_prim_nic[y_nic];
real papp_prim_nic[y_nic];
real pinapp_sec_nic[y_nic];
real papp_sec_nic[y_nic];


### Long Xuyen
real  papp_prim_lx[y_lx] ;// the probabilities of each year being app for prim
real  pinapp_prim_lx [y_lx]; // the probabilities of each year being inapp for prim
real  papp_sec_lx[y_lx] ;// the probabilities of each year being app for sec * prop of the tot sec person years that ar in this year as this will give us the prob needed overall
real papp_sectot_lx;

### Burke
```

```
real pinapp_prim_bur  ; // the probabilities of  being inapp for prim
real papp_prim_bur ;// the probabilities  being app for prim
real pinapp_sec_bur  ;// the probabilities of being inapp for sec
real papp_sec_bur  ;// the probabilities of being app for sec

### Sri Lanka
real pinapp_prim_sr  ; // the probabilities of  being inapp for prim
real papp_prim_sr ;// the probabilities  being app for prim
real pinapp_sec_sr  ;// the probabilities of being inapp for sec
real papp_sec_sr  ;// the probabilities of being app for sec


###KPP1
real papp_prim_all_kpp1;// the probabilities of each serotype  being app for prim
real papp_sec_all_kpp1 ; // the probabilities of each serotype  being app for sec
real papp_eachyear_kpp1 [y_kpp1];// the probabilities of each serotype  being app each year
real pinapp_eachyear_kpp1 [y_kpp1] ;// the probabilities of being inapp each year
real papp_prim_eachyear_kpp1 [y_kpp1];// the probabilities of each serotype  being app each year
real papp_sec_eachyear_kpp1 [y_kpp1] ;// the probabilities of being inapp each year
// on the way to the above we need:
real  papp_prim_kpp1[y_kpp1]  ;// the probabilities of each serotype  being app for prim
real papp_sec_kpp1[y_kpp1]  ; // the probabilities of each serotype  being app for sec
real pinapp_prim_kpp1[y_kpp1] ;// the probabilities of each serotype  being app each year
real pinapp_sec_kpp1[y_kpp1] ;// the probabilities of being inapp each year
real suscprim_kpp1[y_kpp1] ;
real suscsec_kpp1[y_kpp1];


real spi_kpp1 [(y_kpp1-1)];
real ssini_kpp1[(y_kpp1-1)];



real Neachyear_kpp1[y_kpp1];
real pppn_kpp1 [(y_kpp1-1)];

## PR
real papp_prim_pr;
real pinapp_prim_pr;
real papp_sec_pr;
real pinapp_sec_pr;

## PH
real papp_prim_ph;
real pinapp_ph;
real papp_sec_ph;
```

```
// calculating all these probabilities

### Iquitos
for (j in 1:s_iq){
pappprim_iq[j] <- ( lambda_iq[j]*gammaprim) ;// we only need to put proportion that are susceptible to prim or sec here if the data is only both combined- which it isnt here
pinappprim_iq[j] <- (lambda_iq[j]*(1- gammaprim)) ;
pappsec_iq[j] <- ( lambda_iq[j]*gammasec) ;
pinappsec_iq[j] <- (lambda_iq[j]*(1- gammasec)) ;
}



### Lat Am
for (l in 1:y_la){
papp_la[l] <- lambda_la[l]* (((gammaprim *Nprim_la[l])/(Nsec_la[l]+Nprim_la[l]))+((gammasec *Nsec_la[l])/(Nsec_la[l]+Nprim_la[l])) );
pinapp_la[l] <-  lambda_la[l]* ((((1-gammaprim) *Nprim_la[l])/(Nsec_la[l]+Nprim_la[l]))+(((1-gammasec) *Nsec_la[l])/(Nsec_la[l]+Nprim_la[l])) );
}

### Nic
for ( l in 1:y_nic){
papp_prim_nic[l]<-lambda_nic[l]* gammaprim;
papp_sec_nic[l]<-lambda_nic[l]*gammasec ;
pinapp_prim_nic[l]<-lambda_nic[l]* (1-gammaprim );
pinapp_sec_nic[l]<-lambda_nic[l]* (1-gammasec) ;
}

### Long Xuyen
for (l in 1:y_lx){
papp_prim_lx[l] <- ( lambda_lx[l]* gammaprim ) ;// in most cases we would use estimated susceptible proportion
pinapp_prim_lx[l] <- (lambda_lx[l] * (1- gammaprim) ) ;
papp_sec_lx[l]<-((lambda_lx[l]*gammasec*Nsec_lx[l])/(sum(Nsec_lx)));
}
papp_sectot_lx<-sum(papp_sec_lx);

###Burke
pinapp_prim_bur<- (lambda_bur*(1- gammaprim)) ;
papp_prim_bur<- (lambda_bur*gammaprim);
pinapp_sec_bur <- (lambda_bur*(1- gammasec)) ;
papp_sec_bur<-(lambda_bur*gammasec) ;
```

```
###Sri Lanka
pinapp_prim_sr<- (lambda_sr*(1- gammaprim)) ;
papp_prim_sr<- (lambda_sr*gammaprim);
pinapp_sec_sr <- (lambda_sr*(1- gammasec)) ;
papp_sec_sr<-(lambda_sr*gammasec) ;

##KPP1
///// setting the propsusc to prim in first year the same as all the way...
suscprim_kpp1[1]<-Npriminit_kpp1;

for ( k in 1:(y_kpp1-1)){
pppn_kpp1[k]<-1-lambda_kpp1[k];
}

suscprim_kpp1[2]<-Npriminit_kpp1*pppn_kpp1[1];
suscprim_kpp1[3]<-suscprim_kpp1[2]*pppn_kpp1[2];
suscprim_kpp1[4]<-suscprim_kpp1[3]*pppn_kpp1[3];

suscsec_kpp1[1]<-Nsecinit_kpp1;


spi_kpp1[1]<-Npriminit_kpp1*lambda_kpp1[1];
ssini_kpp1[1]<-suscsec_kpp1[1]*pppn_kpp1[1];
suscsec_kpp1[2]<-spi_kpp1[1]+ssini_kpp1[1];

spi_kpp1[2]<-suscprim_kpp1[2]*lambda_kpp1[2];
ssini_kpp1[2]<-suscsec_kpp1[2]*pppn_kpp1[2];
suscsec_kpp1[3]<-spi_kpp1[2]+ssini_kpp1[2];

spi_kpp1[3]<-suscprim_kpp1[3]*lambda_kpp1[3];
ssini_kpp1[3]<-suscsec_kpp1[3]*pppn_kpp1[3];
suscsec_kpp1[4]<-spi_kpp1[3]+ssini_kpp1[3];


for ( k in 1:y_kpp1){

papp_prim_kpp1[k] <- ( lambda_kpp1[k]*gammaprim) ;// we only need to put proportion that are
susceptible to prim or sec here if the data is only both combined- which it isnt here
pinapp_prim_kpp1[k] <- (lambda_kpp1[k]*(1-gammaprim)) ;
papp_sec_kpp1[k] <- (lambda_kpp1[k]*gammasec) ;
pinapp_sec_kpp1[k] <- (lambda_kpp1[k]*(1-gammasec)) ;
}



for ( k in 1:y_kpp1){
```

```
pinapp_eachyear_kpp1[k]<-
(pinapp_prim_kpp1[k]*suscprim_kpp1[k])/(suscprim_kpp1[k]+suscsec_kpp1[k])+
(pinapp_sec_kpp1[k]*suscsec_kpp1[k])/(suscprim_kpp1[k]+suscsec_kpp1[k]);


papp_sec_eachyear_kpp1[k]<-(papp_sec_kpp1[k]*suscsec_kpp1[k]/sum(suscsec_kpp1));
papp_prim_eachyear_kpp1[k]<-(papp_prim_kpp1[k]*suscprim_kpp1[k]/sum(suscprim_kpp1));

}

###PR
pinapp_prim_pr<- (lambda_pr*(1- gammaprim)) ;
papp_prim_pr<- (lambda_pr*gammaprim);
pinapp_sec_pr <- (lambda_pr*(1- gammasec)) ;
papp_sec_pr<-(lambda_pr*gammasec) ;


###Phillipines
papp_prim_ph<- (lambda_ph*gammaprim);
papp_sec_ph<-(lambda_ph*gammasec) ;
pinapp_ph<- lambda_ph*((1- gammaprim)*Nprim_ph/(Nprim_ph+Nsec_ph)+(1-
gammasec)*Nsec_ph/(Nprim_ph+Nsec_ph));
}



model {


int N_la[y_la];
int N_nic[y_nic] ;
int Nsectot_lx;
int Ntot_ph;

lambda_iq ~ beta(1, 1);
lambda_la ~ beta(1, 1);
lambda_nic ~ beta(1, 1);
lambda_lx ~ beta(1, 1);
lambda_bur ~ beta(1, 1);
lambda_sr ~ beta(1, 1);
lambda_kpp1 ~ beta(1, 1);
lambda_pr~ beta(1, 1);
lambda_ph~ beta(1, 1);
## need different Ns for some places:



for ( l in 1:y_la){
```

```
N_la[l]<-(Nsec_la[l])+(Nprim_la[l]);
}

Nsectot_lx<-sum(Nsec_lx);

Ntot_ph<-Nprim_ph+Nsec_ph;

// the estimates of the things we have data for given the proportions above.

### Iquitos
appsec_iq   ~  binomial(Nsec_iq, pappsec_iq) ;
appprim_iq  ~  binomial(Nprim_iq, pappprim_iq) ;
inappsec_iq   ~  binomial(Nsec_iq, pinappsec_iq) ;
inappprim_iq   ~  binomial(Nprim_iq, pinappprim_iq) ;


# ### Lat Am
app_la  ~  binomial(N_la, papp_la);
inapp_la ~  binomial(N_la, pinapp_la);

## Nicaragua
app_prim_nic ~  binomial(Nprim_nic, papp_prim_nic);
inapp_prim_nic ~  binomial(Nprim_nic, pinapp_prim_nic);
app_sec_nic ~  binomial(Nnotprim_nic, papp_sec_nic);
inapp_sec_nic ~  binomial(Nnotprim_nic, pinapp_sec_nic);

### Long Xuyen
app_prim_lx  ~  binomial(Nprim_lx, papp_prim_lx);
inapp_prim_lx ~  binomial(Nprim_lx, pinapp_prim_lx);
app_sectot_lx  ~  binomial(Nsectot_lx, papp_sectot_lx);

### Burke

app_prim_bur   ~  binomial(Nprim_bur, papp_prim_bur) ;
app_sec_bur  ~  binomial(Nsec_bur, papp_sec_bur) ;
inapp_sec_bur   ~  binomial(Nsec_bur, pinapp_sec_bur) ;
inapp_prim_bur   ~  binomial(Nprim_bur, pinapp_prim_bur) ;

### Sri Lanka

app_prim_sr   ~  binomial(Nprim_sr, papp_prim_sr) ;
app_sec_sr ~  binomial(Nsec_sr, papp_sec_sr) ;
inapp_sec_sr   ~  binomial(Nsec_sr, pinapp_sec_sr) ;
inapp_prim_sr   ~  binomial(Nprim_sr, pinapp_prim_sr) ;

#KPP1
for ( k in 1:y_kpp1){
app_prim_eachyear_kpp1[k] ~  binomial(Nprim_kpp1, papp_prim_eachyear_kpp1[k]) ;
```

```
app_sec_eachyear_kpp1[k] ~  binomial(Nsec_kpp1, papp_sec_eachyear_kpp1[k]) ;
inapp_eachyear_kpp1[k]  ~  binomial(Ntoteachyear_kpp1[k], pinapp_eachyear_kpp1[k]) ;
}

##PR

app_prim_pr   ~  binomial(Nprim_pr, papp_prim_pr);
app_sec_pr ~  binomial(Nsec_pr, papp_sec_pr);
inapp_sec_pr   ~  binomial(Nsec_pr, pinapp_sec_pr);
inapp_prim_pr   ~  binomial(Nprim_pr, pinapp_prim_pr);

## Phillipines

app_prim_ph   ~  binomial(Nprim_ph, papp_prim_ph) ;
app_sec_ph ~  binomial(Nsec_ph, papp_sec_ph) ;
inapp_ph   ~  binomial(Ntot_ph, pinapp_ph) ;



}
'
```

Data from the papers

```
asymptomatic_dat_cohorts<- list(s_iq = 2,
                        appprim_iq=c(28,35),
                        inappprim_iq = c(109,168),
                        appsec_iq=c(16,54),
                        inappsec_iq = c(107,370),

                        Nprim_iq=c(371, 345),
                        Nsec_iq=c(353, 599),


                        y_la=4 ,
                        app_la=c(5,6,1,6)   ,
                        inapp_la = c(15,8,8,19),
                        Nprim_la=c( 200, 88, 340, 139),
                        Nsec_la=c( 249, 1086, 353,133),

                        y_nic=4,
                        app_prim_nic=c(5, 24, 7, 19),
                        inapp_prim_nic=c(98, 148, 80, 123),
                        app_sec_nic=c(10, 40, 4, 41),
                        inapp_sec_nic=c(195, 262, 143, 117),
                        Nprim_nic=c(1162, 1408, 1382, 1547),
                        Nnotprim_nic=c(1667, 2018, 1774, 1794),
```

```
y_lx= 4,
app_prim_lx=c(31,39,46,73),
inapp_prim_lx = c(163,220,129,252),
app_sectot_lx=123,
Nprim_lx=c(1617, 2158, 2215, 2389),
Nsec_lx=c(444, 858, 790, 562),


inapp_prim_bur=c(43),
inapp_sec_bur = c(47),
app_prim_bur=c(4),
app_sec_bur=c(9),
Nprim_bur=c(747),
Nsec_bur=c(1010),

inapp_prim_sr=c(20),
inapp_sec_sr=c(20),
app_prim_sr=c(15),
app_sec_sr=c(12),
Nprim_sr=c(358),
Nsec_sr=c(441),

y_kpp1=4,
app_prim_eachyear_kpp1=c(6,2,5,0),
app_sec_eachyear_kpp1=c(27, 24,80,38),
inapp_eachyear_kpp1=c(81, 77, 103, 85),
Ntoteachyear_kpp1=c(2023, 2021, 2039, 2007),
Nprim_kpp1=c(1500), Nsec_kpp1=c(2304),
Npriminit_kpp1=c(423), Nsecinit_kpp1=c(847),

app_prim_pr=c(1),
inapp_prim_pr=c(2),
app_sec_pr=c(8),
inapp_sec_pr=c(8),
Nprim_pr=c(26),Nsec_pr=c(144),

app_prim_ph=c(4),
app_sec_ph=c(9),
inapp_ph=c(61),
Nprim_ph=c(87),
Nsec_ph=c(790)




)
```

```
fit_asy_cohorts<-stan(model_code=asymptomatic_code_cohorts, data=asymptomatic_dat_cohorts,
iter=6000,chains=4)
sasy_cohorts<-extract(fit_asy_cohorts)
```
